# Supplementary material for: Successional Shifts in Soil C:N:P stoichiometry and its environmental controls during forest restoration in Huize County, Qujing, Yunnan Province
Source: PLoS One. 2026 Aug 21;21(8):e0356261. doi: 10.1371/journal.pone.0356261 (PMC13496410; doi:10.1371/journal.pone.0356261)
Supplement: Supplementary file 2 [file pone.0356261.s004.zip › Data and Code/RF/mixed conifer–broadleaf forest/figures/PDP_C.P_s.TP.pdf]

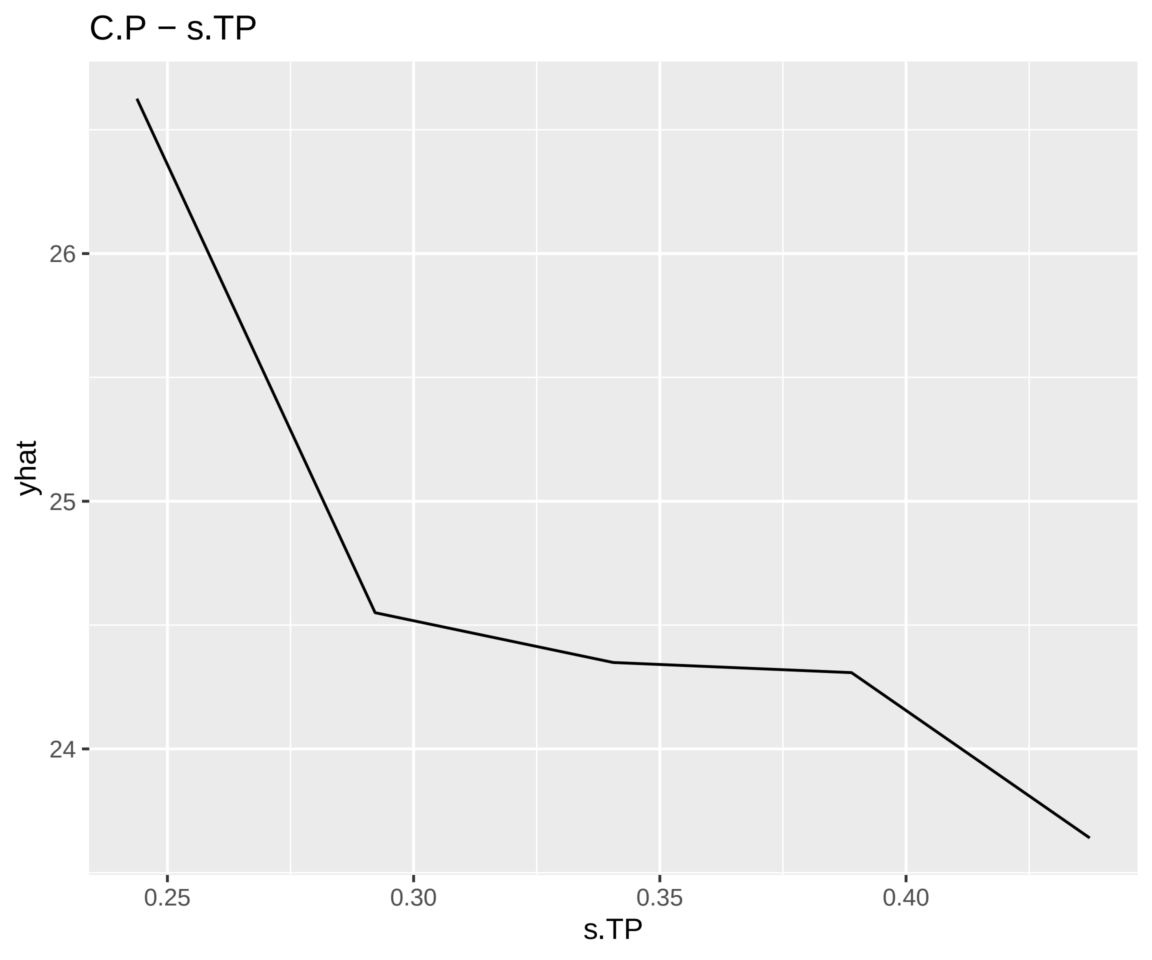
C.P – s.TP
yhat
26
25
24
0.25
0.30
0.35
0.40
s.TP
